# Mental Health Literacy Scale Arabic Version: A Validation Study Among Saudi University Students

**DOI:** 10.3389/fpsyt.2021.741146

**Published:** 2021-09-27

**Authors:** Elham Alshehri, Dalyal Alosaimi, Ebtisam Rufaidi, Nesreen Alsomali, Regie Tumala

**Affiliations:** ^1^Nursing Education Department, Second Cluster, Ministry of Health, Riyadh, Saudi Arabia; ^2^Community and Mental Health Nursing Department, College of Nursing, King Saud University, Riyadh, Saudi Arabia; ^3^Medical-Surgical Nursing Department, College of Nursing, King Saud University, Riyadh, Saudi Arabia; ^4^Non-invasive Cardiology Department, Armed Forces Hospital Southern Region, Abha, Saudi Arabia

**Keywords:** mental health, mental health literacy, psychometric evaluation, university students, Saudi Arabia

## Abstract

Mental health problems significantly affect students' university education. However, studies about mental health literacy (MHL) among Saudi university students are very limited. The two-fold objective of this study was to validate an Arabic version of the Mental Health Literacy Scale (MHLS) and assess the MHL of university students. The study participants involved 339 female students. Psychometric evaluation of the MHLS–Arabic version was conducted, and correlation and regression analyses were performed. The scale was successfully cross culturally adapted and found to be valid and reliable. The highest MHL indicator was the students' perception of confidence in using a computer or telephone to inquire about mental illness data. Conversely, the lowest MHL indicator was the student's disagreement with the notion that mental infirmity is not an actual medical challenge. Marital status, college attended, and academic level were found to have statistically significant effects on the MHL of university students. The Arabic version of the MHLS validly and reliably assessed MHL. This work adds to existing evidence for assessing MHL and can help administrators formulate better strategies to improve the MHL of university students.

## Introduction

The challenges associated with mental health significantly impinge on academic performance (1, 2) and increase tertiary education dropout rates (3). Students are often attracted to study in universities of their chosen programs. However, they are rarely adequately prepared and acquainted with the realities associated with the effect of university life on their physical, emotional, spiritual, and mental well-being. As cited by Nguyen Thai and Nguyen (4) from the World Health Organization (5), approximately 450 million individuals have mental disorders, and these conditions commonly arise during adolescence and young adulthood. Common mental health conditions reported among Saudi adolescents include: intellectual disability (most common), anxiety disorders, attention deficit hyperactivity disorder, autistic spectrum disorders, seizure disorders, mood disorders, school refusal, enuresis and encopresis, psychosis, tic disorder, selective mutism, stuttering, adjustment disorder, oppositional defiant disorder, avoidant personality traits, V-code diagnoses, conduct disorder, conversion disorder, or sexual abuse (6). Specifically, students experience increased levels of psychological and academic distress, succumb to the pressures of studying, and further encounter stressors from family instability and dysfunction, substance misuse, and mental disorders (7–9). All these developments can result in the students' failure to complete their university education (7–9). Moreover, students who are exposed to mental health risks including physical abuse and bullying are both associated with higher odds of having more frequent anxiety and depression symptoms and having poorer academic performance (10). Additionally, having mental health problems during adolescence poses higher risks into adulthood. For example, prevalence of depressive disorder in adolescence has strong risk of recurrence during adulthood which was reported in different countries, including Bahrain (11), Canada (12), and United States of America (USA) (13). In addition, a study among Saudi adult patients revealed that high proportions of the participants had diagnosable mental illness (e.g., anxiety, bipolar, depressive, and psychotic disorders) and sought treatment from faith healers believing that their disorders were attributed to faith-based reasons such as evil eye and magic (14). Nonetheless, reports show that mental health services are commonly available for students in universities across many countries, including Saudi Arabia (6).

According to Kutcher et al. (15), the high school-to-university transition for young people is a valuable time to augment their mental health knowledge. An estimated 50% of young adults aged 18–24 years old are undertaking university education. Mental health literacy (MHL) interventions targeting these young adults represent an opportunity to implement programs to improve MHL (16). According to Jorm et al. (17), MHL refers to the knowledge and attitudes related to mental well-being that help to detect, manage, and deter mental health problems. The definition of MHL stresses the role of recognizing mental health challenges and aid-seeking behaviors for the management and prevention of mental health problems by young adults and the immediate people in their lives, such as family members, teachers, and friends (4, 16).

Numerous studies in Eastern and Western nations have examined the impact of education among university nursing students with regards to their knowledge and approaches on mental health and illness (18–23). Other studies involving adolescents in Ireland (24) and in Norway (25), young adults in Portugal (26), and among employees in Germany (27) reported the influence of sociodemographic variables (e.g., age, gender, education, place of residency, proximity to people presenting mental disorders, and Internet access) on MHL. In addition, media as well as culture has been considered as an influential source of information and opinion on mental health worldwide, including Saudi Arabia (6, 28). A study in United Kingdom (UK) implied that unidentified Internet resources should be modeled to aid college learners to become more informed about mental well-being and easily seek help and proper support (29). Additionally, a cross-cultural study by Liu et al. (21) revealed a significant need for clinical practice procedures to control mental challenges among American nursing students and precise teaching on safe alcohol use practices among Chinese nursing students. Hence, there is evidence showing that sociodemographic characteristics of study participants affect their MHL.

According to Mahfouz et al. (30), MHL has been studied mainly in developed countries [e.g., (31–33)], with one study conducted in the Arab region [i.e., (34)]. In Saudi Arabia, the public was reported to show decreased levels of MHL, negative attitudes, and stigmatization of mental disorders (35). The undertaking of additional efforts and interventions was suggested to educate the public about the psychobiological underpinnings of mental illnesses and the value of effective treatments. Concurrently, another study using an Arabic questionnaire about public perception of mental health indicated that the degree of MHL of Saudi university students was intermediate and necessitated the implementation of health education programs to change the students' attitudes regarding mental illness (30). Challenges persist because mental health among young adults has never been considered a high priority in Saudi Arabia. Furthermore, Liu et al. (21) suggested a crucial need to establish a culturally sensitive curriculum. In this curriculum, the different beliefs about intercessions to manage mental illnesses are acknowledged among health professionals.

The literature shows that health literacy studies are limited among female population in Saudi Arabia (36). O'Connor et al. (37) indicated there are substantial limitations in measuring MHL and emphasized the need to evaluate the psychometric robustness of the Mental Health Literacy Scale (MHLS). In particular, research concerning the culturally sensitive assessment of MHL among Saudi university students are few. Moreover, no investigation has been conducted using an Arabic version of the MHLS. Instead of creating new scales for Saudi Arabia, studies have recommended implementing already existing measures and evaluating these measures in various languages and cultures (38, 39). However, these measures must be translated and validated according to strict methodology to ascertain the semantic and scientific quality of the content in the new cultural version of the scale (38–40). Thus, the two-fold objective of this work was to culturally adapt and validate an Arabic version of the MHLS, and explore Saudi university students' MHL levels.

## Materials and Methods

### Research Design

This study used a correlational and cross-sectional design. This work also adhered to Strengthening the reporting of observational studies in epidemiology (STROBE) checklist for cross-sectional studies. The authors did not include any discussion related to COVID-19 implications since the data of the study were collected before the pandemic.

### Sample and Setting

This study was conducted in three colleges at a premier governmental university in Riyadh, Saudi Arabia among undergraduate students between December 2018 and April 2019. Convenience sampling was used to obtain greater involvement among university students as defined by the inclusion and exclusion criteria. The inclusion criteria of this study were as follows: Saudi female students who were officially registered for the Academic Year 2018–2019, who were bonafide students from the three colleges of the university, who were available at the time data were collected, and who willingly and voluntarily participated. Only female university students were included in this investigation because of gender segregation in public places including state universities as the cornerstone of the Saudi interpretation of Islam (41–44). Furthermore, the female and male campuses are separated in this setting. Hence, male students were excluded due to challenges (e.g., a married researcher needs her husband to be around during data collection in the male campus) in recruiting them to participate in the study. Even though changes have been signaled to lift some restrictions toward Saudi women, they must have someone who has the power to make a range of critical decisions on their behalf (a male guardian, father or husband). This development has led to extensive separate public spaces that are only for women (42–45). Thus, accessibility to the male university students was difficult. Future studies are needed to assess the MHL of male university students. The exclusion criteria of the study were as follows: students who are Saudi males, enrolled in an internship practicum program, non-Saudi students, under 18 years old, unwilling to participate at the time data were collected, and participants in the pretest. We recruited 400 female students, and 351 surveys were retrieved (response rate: 88%).

### Ethical Considerations

Ethical approval from the Institutional Review Board (IRB) at the College of Medicine in King Saud University (Research Project No.: E-18-3617) and permissions from the deanships of the three colleges where the participants were enrolled were obtained before conducting the study. Trained research assistants recruited prospective participants in their free time. The research assistants approached the participants individually or in groups as the situation arose. The objectives were explained, and informed consent was provided by those who voluntarily participated in the survey. Anonymity was explained and secured by omitting the participants' names in the questionnaires. The confidentiality of the responses was assured by using only the codes of responses to process and analyze the survey results. Respondents were informed that they had the freedom to withdraw from the research study without any consequence. Questionnaires were distributed to those who willingly and voluntarily consented to participate in the study. Data were obtained from the completed surveys.

### Instrumentation

#### Demographic Data Questionnaire

Demographic background data included age; marital status; college attended; academic level; place of residence; and personal history, family history, and relative history of mental disorder.

#### Mental Health Literacy Scale

Permission to translate and use the MHLS was granted by the copyright holder. The MHLS has 35 items (46) that were either adapted or customized for equivalency in this study. The original English version of the scale had six factor structures: (1) capacity to identify disorders (eight items), (2) acquaintance with where to obtain data (four items), (3) awareness of risk factors and causes (two items), (4) familiarity with self-treatment (two items), (5) awareness of the availability of professional help (three items), and (6) thoughts that support identification or effective aid-seeking behavior (16 items). The total score of the scale was obtained by summing all 35 items. Questions with a four-point Likert scale were rated as follows: very unlikely/unhelpful (1), unlikely (2), likely (3), and very likely (4). Questions with a five-point Likert scale were rated as follows: strongly disagree/definitely unwilling (1), disagree/probably unwilling (2), neither agree/disagree or neither unwilling/willing (3), agree/probably willing (4), and strongly agree/definitely willing (5). Questions 10, 12, 15, and 20–28 were reversely scored before the data were statistically analyzed. In the original scale, the highest score was 160, and the lowest was 35. In this study, responses with four-point Likert were recoded to five-point Likert responses in order to compute the overall mean score of MHL. Questions with high mean scores were interpreted as indicative of having high level MHL.

#### Translation, Cross-Cultural Adaptation, and Validity of the MHLS

Guidelines for the interpretation, cross-cultural adaptation, and legalization of the MHLS into the Arabic language using five stages were adapted (47). The production of the Arabic version followed five stepwise stages of translation, synthesis, back translation, review of experts, and pretesting and pilot study. In Stage 1, the MHLS was translated by a translation office in Riyadh, Saudi Arabia and two bilingual expert English-to-Arabic translators double-checked the translated English items to Arabic items. The aim at this phase (Stage 1) was to attain equivalency of the English items into Arabic so as to attain cross-cultural adaptation. Stage 2 involved the evaluation and production of Arabic translations by two bilingual local Arabic experts who assessed the semantic, idiomatic, and theoretical correspondence of the Arabic translations. Minor discrepancies were resolved, and agreement on the Arabic translation was reached. Stage 3 entailed back translation of the Arabic version to English by two expert Arabic-to-English translators. Stage 4 denoted the analysis of the Arabic translation and back-translated versions by two bilingual experts for consensus confirmation of the pre-final version. Face validation according to consensus agreement of the pre-final Arabic version was obtained.

An additional procedure was adopted to ensure the validity of the Arabic version. The said version of the survey was subjected to additional validity assessments using the item-level content validity index (I-CVI) wherein every item was rated for relevance. The scale-level content validity index (S-CVI) involved computing the quantity of items rated as quite or very relevant by every rater (48). Six item experts inspected and assessed the independent items. The validation tests produced an average of 1.0 for the I-CVI and an overall S-CVI of 1.0, thereby suggesting that the items in the instrument are essential and relevant. Overall, the MHLS—Arabic version was satisfactory and had excellent content legality. Stage 5 required the pretesting of MHLS—Arabic version with 30 Saudi university student participants. The university students evaluated the scale's clarity and ease in completion. Feedback from the pretest participants indicated that the questionnaire was moderately easy to very easy to complete. They further reported that the instrument was easy to read and understand, and that they finished the survey between 12 and 15 minutes.

### Data Analyses

Descriptive statistics were calculated (e.g., median, mean, and standard deviation). Spearman's correlation coefficient was used to measure the association between variables because the data did not follow a normal distribution. Furthermore, regression analysis was employed to determine the factors that had significant effects on MHL. Factor analysis was performed on the 35 items of MHLS—Arabic version. Significant findings were inferred if *p* < 0.05. The data were processed using IBM SPSS Statistics for Windows v.21 (Armonk, New York: IBM Corp. 2012).

## Results

### Exploratory Factor Analysis

Exploratory factor analysis (EFA) results are shown in Table 1. Data screening for the missing values of the 351 cases and reverse coding of the responses were performed. Testing for multivariate outliers was conducted using the Mahalanobis distance criteria of $\chi_{\left(35\right)}^{2}$ > 66.62. Thirty-five degrees of freedom was equivalent to the number of items to be tested (49). Twelve cases had Mahalanobis $\chi_{\left(35\right)}^{2}$ > 66.62 and were deleted from the dataset. Hence, 339 cases were included in the subsequent analytic procedures. Multicollinearity and singularity diagnostics were undertaken using tolerance values, squared multiple correlations, and the condition index. Neither multicollinearity nor singularity existed for any of the variables in the dataset. Most variables were negatively skewed. However, examination of linearity through scatter plots suggested no evidence of curvilinearity. Hence, the dataset passed the linearity test (49).

**Table 1:** Results of exploratory factor analysis and reliability tests of Mental Health Literacy Scale (MHLS)—Arabic version (*N* = 339).

**Variables**	**Factor** **loading**	**Reliability tests**		
		**Corrected** **item-total** **correlation**	**Cronbach's** **alpha if item** **is deleted**	**Overall** **Cronbach's** **alpha**
Q24	0.70	0.67	0.83	
Q21	0.69	0.65	0.83	
Q27	0.69	0.63	0.83	
Q28	0.69	0.63	0.83	
Q22	0.66	0.61	0.83	
Q26	0.62	0.59	0.84	
Q23	0.58	0.55	0.84	
Q25	0.47	0.41	0.85	
**Factor 1: Attitude toward mental illness**				**0.85**
Q31	0.78	0.76	0.86	
Q29	0.73	0.70	0.86	
Q33	0.72	0.67	0.87	
Q35	0.72	0.71	0.86	
Q32	0.72	0.71	0.86	
Q30	0.69	0.68	0.87	
Q34	0.60	0.53	0.88	
**Factor 2: Attitude to someone with mental illness**				**0.89**
Q7	0.67	0.59	0.75	
Q5	0.63	0.56	0.76	
Q11	0.55	0.50	0.77	
Q1	0.53	0.46	0.77	
Q4	0.52	0.46	0.77	
Q6	0.50	0.45	0.77	
Q2	0.48	0.45	0.77	
Q3	0.46	0.43	0.78	
Q12	0.45	0.42	0.78	
**Factor 3: Recognition of disorders**				**0.79**
Q17	0.65	0.63	0.59	
Q19	0.64	0.58	0.62	
Q16	0.54	0.51	0.66	
Q18	0.48	0.34	0.76	
**Factor 4: Mental illness information-seeking**				**0.72**

Principal axis factoring using Varimax rotation was executed on the 35 items. Principal axis factoring was selected given its capability of computing factors that are compatible with factor analysis (50). As a method of orthogonal rotation, Varimax rotation was utilized because it maximizes the dispersion of loadings within factors, thereby resulting in loading fewer variables highly onto each factor and in clusters of factors that can be interpreted more easily (51). Examination of the correlation coefficients indicated a sufficient number of *r* > 0.3. Thus, the items displayed factorability. The Kaiser–Meyer–Olkin measure of sampling adequacy = 0.848, and thus the sample size was adequate and meritorious (52). Bartlett's Test of Sphericity [$\chi_{\left(595\right)}^{2}$ = 4129.33, *p* < 0.001] showed that the information set was excellent. The initial factor solution yielded eight factors with eigenvalues >1 and collectively elucidated 57.91%. However, Factors 5, 6, 7, and 8 had inadequate factor loadings (<0.45) and were challenging to interpret as subgroups.

Further examination of the Scree plot suggested four distinct factors. Hence, the factor solution was repeated by setting the four factors to be extracted. The four factors that loaded collectively explained 44.00% of the variances in the solution. Variables with factor charging ≥0.45 were included in each factor. Comrey and Lee (53) suggested a high factor loading of a variable indicates a pure measure of the factor. The factor loadings of the variables included in the final solution ranged between 0.45 and 0.78, and those outcomes were interpreted as fair to excellent (53).

The final solution with the variables, factor loading, corrected item–total correlation, alpha if the item was deleted and the subscale Cronbach's alpha rounded to two decimals was applied (Table 1). Factor 1 had a Cronbach's alpha = 0.85 with an individual corrected item-total correlation ranging between 0.41 and 0.67. Collectively, the subscale was considered to have adequate reliability and yielded eight items more likely to describe the attitude toward mental illness. Factor 2 converged with seven items that were more likely to collectively describe the attitude toward someone with mental illness, with a Cronbach's alpha = 0.89 and an individual corrected item-total correlation ranging between 0.53 and 0.76. These outcomes collectively suggested that the subscale had adequate reliability. Factor 3 converged with nine items having Cronbach's alpha = 0.79 with an individual corrected item-total correlation ranging between 0.42 and 0.59. Collectively, the subscale was considered to have adequate reliability and was labeled as the recognition of disorders. Finally, Factor 4 converged with four items with Cronbach's alpha = 0.72 and an individual corrected item-total correlation ranging between 0.34 and 0.63. Collectively, the subscale was considered to have adequate reliability and was labeled as mental illness information seeking.

In this study, the wording of the variables that were loaded in Factors 1–4 more likely described the concept of mental illness than mental health. Hence, the subscales were labeled according to the predominant themes in most variables with a predisposition toward the concept of mental illness. Overall, the EFA for this dataset yielded four subscales with sufficient factor loadings and reliability.

### Demographic Characteristics

The demographic characteristics of the participants (Table 2) indicated the following. Most participants were aged between 18 and 20 years old (54.57%), and almost all were single (90.56%). Many of the respondents were from the Colleges of Medicine (39.82%) and Nursing (34.51%), and a few were from the College of Pharmacy (25.66%). Many respondents were from Level 4 (42.77%). Most participants lived in the northern part inside the Riyadh region (44.25%).

**Table 2:** Demographic characteristics of female Saudi university students (*N* = 339).

**Demographic characteristics**		***n***	**%**
Age	18–20	185	54.57
	21–25	129	38.05
	26–28	25	7.37
Marital status	Single	307	90.56
	Married	28	8.26
	Divorced	4	1.18
College attended	Medicine	135	39.82
	Nursing	117	34.51
	Pharmacy	87	25.66
Academic level	Level 3	84	24.78
	Level 4	145	42.77
	Level 5	43	12.68
	Level 6	67	19.76
Place of residence	Inside Riyadh	318	93.81
	Outside Riyadh	21	6.19
Inside Riyadh	North	150	44.25
	South	49	14.45
	East	81	23.89
	West	38	11.21
Outside Riyadh	North	2	0.59
	South	1	0.29
	East	4	1.18
	West	14	4.13

### Mental Health Disorder History of Saudi University Students, Their Families, and Relatives

Most of the university students reported no personal history of mental disorder but 40 of them reported a personal history of psychiatric disorders (Table 3). Meanwhile, the reported mental disorders of the participants were mostly undiagnosed. Most participants stated that their family had no history of psychiatric disorders. However, 55 of the respondents reported a history of mental disorders with 40 who reported being diagnosed but were unsure about the duration. Most participants stated that their relatives had no history of mental disorders and 60 of them reported that most of their relatives had a history of diagnosed mental disorders. However, most of the 60 participants were unsure about the duration of the mental disorders of their relatives.

**Table 3:** Mental health disorder history of Saudi university students, their families and relatives (*N* = 339).

**Items**	**Sub-items**	***n***	**%**
Personal mental health disorder	No	299	88.20
	Yes	40	11.80
If yes (*n* = 40)	Diagnosed	3	7.50
	Undiagnosed	11	27.50
	Unsure	26	65.00
If yes, since (*n* = 40)	More than 3 years	3	7.50
	1–3 years	11	27.50
	<1 year	5	12.50
	Unsure	21	52.50
Mental health disorder history in family	No	284	83.78
	Yes	55	16.22
If yes (*n* = 55)	Diagnosed	40	72.73
	Undiagnosed	13	23.64
	Unsure	2	3.63
If yes, since (*n* = 55)	More than 3 years	19	34.55
	1–3 years	7	12.73
	Less than 1 year	4	7.27
	Unsure	25	45.45
Mental health disorder history among relatives	No	279	82.30
	Yes	60	17.70
If yes (*n* = 60)	Diagnosed	43	71.67
	Undiagnosed	13	21.67
	Unsure	4	6.66
If yes, since (*n* = 60)	More than 3 years	22	36.67
	1–3 years	5	8.33
	<1 year	32	53.33
	Unsure	1	1.67

### Personal Mental Health Disorder History of Saudi University Students

A total of 339 university students participated in the study (Table 4). The university students reported a personal history of mental disorders such as schizophrenia (0.88%), shared psychiatric disorder (0.88%), psychiatric disorder due to another medical condition (0.59%), dissociative identity disorder (0.29%), and postpartum psychiatric disorder (0.29%). They also described having neurotic mental disorders such as depressive neurosis (7.37%), anxious neurosis (3.83%), eating disorder (1.77%), post-traumatic stress disorder (0.59%), postpartum depression (0.59%), and obsessive-compulsive disorder (0.29%). Furthermore, they claimed to have psychosomatic mental disorders which arose from psychological source with no clear biological or medical basis such as acne (3.54%), headache (2.95%), arrhythmia (1.47%), irritable colon (1.47%), allergy (1.18%), bronchial asthma (0.88%), gastric ulcer (0.88%), diabetes mellitus (0.59%), and herpes ulcer (0.29%).

**Table 4:** Personal history of mental health disorders of Saudi university students (*N* = 339).

**Category**	**Mental health disorder**	***n***	**%**
Psychosis	Schizophrenia	3	0.88
	Shared psychiatric disorder	3	0.88
	Psychiatric disorder due to another medical condition	2	0.59
	Dissociative identity disorder	1	0.29
	Postpartum psychosis	1	0.29
Neurosis	Depressive neurosis	25	7.37
	Anxious neurosis	13	3.83
	Eating disorder	6	1.77
	Post-traumatic stress disorder	2	0.59
	Postpartum depression	2	0.59
	Obsessive compulsive disorder	1	0.29
Psychosomatic	Acne	12	3.54
	Headache	10	2.95
	Arrhythmia	5	1.47
	Irritable colon	5	1.47
	Allergy	4	1.18
	Bronchial asthma	3	0.88
	Gastric ulcer	3	0.88
	Diabetes mellitus	2	0.59
	Herpes ulcer	1	0.29

### Mental Health Literacy of Saudi University Students

Regarding the perceived MHL of participants (Table 5), the overall mean score was 3.43 from 5 (*SD* = 1.12). The overall mean score was the compilation of correct answers of the participants which indicated high levels of MHL. The attitude toward mental illness factor mean score was 3.89 (*SD* = 0.87), as the factor perceived to have the highest level of MHL. The mental illness information-seeking factor mean score was 3.85 (*SD* = 0.91) and the attitude to someone with mental illness factor mean score was 3.10 (*SD* = 1.07). The lowest level of MHL was reported on the recognition of disorders factor (*M* = 2.86, *SD* = 0.82). Furthermore, the highest indicator was Item 17 (*M* = 4.76, *SD* = 0.65), an outcome which demonstrated that the participants were certain about using a computer or telephone to obtain data about mental disorders. However, the lowest was Item 22 (*M* = 1.65, *SD* = 0.87). Thus, participants disagreed with the assertion that mental challenges are actual medical disorders.

**Table 5:** Mental health literacy of Saudi university students (*N* = 339).

**Mental health literacy indicators**	***Mean***	***SD***
**Factor 1: Attitude toward mental illness**	**3.89**	**0.87**
21. A mental illness is a sign of personal weakness. ^*r*^	4.70	0.59
22. A mental illness is not a real medical illness. ^*r*^	1.65	0.87
23. People with a mental illness are dangerous. ^*r*^	3.64	0.90
24. It is best to avoid people with a mental illness so that you don't develop this problem. ^*r*^	4.21	0.89
25. If I had a mental illness, I would not tell anyone. ^*r*^	3.36	1.03
26. Seeing a mental health professional means you are not strong enough to manage your own difficulties. ^*r*^	4.48	1.09
27. If I had a mental illness, I would not seek help from a mental health professional. ^*r*^	4.61	0.70
28. I believe treatment for a mental illness, provided by a mental health professional, would not be effective. ^*r*^	4.45	0.87
**Factor 2: Attitude to someone with mental illness**	**3.10**	**1.07**
29. How willing would you be to move next door to someone with a mental illness?	2.88	1.02
30. How willing would you be to spend an evening socializing with someone with a mental illness?	3.61	0.86
31. How willing would you be to make friends with someone with a mental illness?	3.42	1.23
32. How willing would you be to have someone with a mental illness start working closely with you on a job?	3.55	1.06
33. How willing would you be to have someone with a mental illness marry into your family?	2.36	0.99
34. How willing would you be to vote for a politician if you knew they had suffered a mental illness?	2.39	1.25
35. How willing would you be to employ someone if you knew they had a mental illness?	3.48	1.09
**Factor 3: Recognition of disorders**	**2.86**	**0.82**
1. If someone became extremely nervous or anxious… to what extent do you think it is likely they have Social Phobia?	3.00	0.71
2. If someone experienced excessive worry… to what extent do you think it is likely they have Generalized Anxiety Disorder?	3.27	0.67
3. If someone experienced a low mood for two or more weeks… to what extent do you think it is likely they have Major Depressive Disorder?	2.79	0.86
4. To what extent do you think it is likely that Personality Disorders are a category of mental illness?	2.97	0.77
5. To what extent do you think it is likely that Dysthymia is a disorder?	3.09	0.77
6. To what extent do you think it is likely that the diagnosis of Agoraphobia…?	2.82	0.98
7. To what extent do you think it is likely that the diagnosis of Bipolar Disorder…?	2.76	0.97
11. To what extent do you think it is likely that Cognitive Behavior Therapy (CBT)…?	3.30	0.81
12. To what extent… mental health professional to break confidentiality: if you are at immediate risk of harm to yourself or others? ^*r*^	1.73	0.88
**Factor 4: Mental illness information-seeking**	**3.85**	**0.91**
16. I am confident that I know where to seek information about mental illness.	3.39	1.03
17. I am confident using the computer or telephone to seek information about mental illness.	4.76	0.65
18. I am confident attending face to face appointments to seek information about mental illness (e.g., seeing the GP).	3.45	1.18
19. I am confident I have access to resources (e.g., GP, internet, friends) that I can use to seek information about mental illness.	3.79	0.89
**Overall mental health literacy**	**3.43**	**1.12**

r *Reverse scored questions*.

### Relationship Between the Demographic Characteristics of Saudi University Students and Their MHL

In the regression analysis (Table 6), age (*r* = 0.10, *p* = 0.85) exhibited a positive but non-significant correlation with MHL. Meanwhile, marital status (*r* = 0.19, *p* = 0.04), college attended (*r* = 0.06, *p* = 0.03), and academic level (*r* = 0.03, *p* = 0.03) showed a positively significant correlation with MHL. Place of residence (*r* = −0.06, *p* = 0.06), indicated a negative but non-significant relationship with MHL. Furthermore, regression analysis revealed that marital status (ß = 0.19, 95% CI = −0.60, 1.79), college attended (ß = 0.15, 95% CI = −0.05, 0.53), and academic level (ß = 0.08, 95% CI = −0.20, 0.41) had effects on the MHL among female university students.

**Table 6:** Relationship between the demographic characteristics of university students and their mental health literacy (*N* = 339).

**Variables**	***r***	**Unstandardized coefficients**		**Standardized coefficients**	***t***	***p*-value**	**(95% Confidence interval)**	
		***B***	**Standard error**	**Beta**			**Upper**	**Lower**
Age	0.10	0.31	0.31	0.13	1.01	0.85	−0.32	0.94
Marital status	0.19	0.60	0.58	0.19	1.02	0.04^*^	−0.60	1.79
College attended	0.06	0.24	0.14	0.15	1.68	0.03^*^	−0.05	0.53
Academic level	0.03	0.10	0.15	0.08	0.70	0.03^*^	−0.20	0.41
Place of residence	−0.06	1.49	0.62	0.46	2.40	0.06	−0.22	2.77

*Dependent variable: mental health literacy*.

* *Significance level at p < 0.05*.

## Discussion

This study culturally adapted and validated the 35 items of the MHLS as a 28-item Arabic version. The developed version revealed acceptable and excellent content and scale validity, as well as sufficient factor loadings and reliability. Jorm et al. (17) introduced the vignette-interview design as the most common approach to assess MHL. This technique involved the identification of a disorder presented within a vignette. Since then, developing reliable measures to assess MHL has been challenging (54). Similarly, the wording of the variables that were loaded in Factors 1–4 of the MHLS—Arabic version more likely described the concept of mental illness than mental health. O'Connor et al. (37) identified several scale-based measures that failed to assess all aspects of MHL because they lacked psychometric robustness. Recently, O'Connor and Casey (46) created the MHLS as the first psychometrically-sound measure to assess all attributes of Jorm's MHL model. The EFA conducted in this work was the first attempt to evaluate the psychometric properties of the MHLS—Arabic version and yielded four subscales with sufficient factor loadings and reliability. However, the MHLS—Arabic version might not assess all aspects of the MHL of university students because seven items were deleted from the scale. As such, future MHL studies should evaluate the psychometric-robustness of the MHLS—Arabic version with all aspects of MHL included.

Most participants were aged between 18 and 20 years old. Almost all of them were single, and many were in their second year at university (Level 4). Furthermore, most respondents lived in the northern part inside Riyadh City. As most of the participants in this study were in their second year (Level 4), this condition differs from other studies with participants in their first year at university (15, 29, 46). This work is similar to a survey that found that most participants were females aged 15–21 years old residing in an urban area (16, 22, 29, 46). Additionally, the present research is comparable to other investigations in Portugal (26) and Saudi Arabia (30) for which most participants attending college and university were single.

However, unlike the results in this study, being born outside of Norway was considerably associated with elevated stress levels, as well as being from an economically disadvantaged family (25). In this work, most participants who reported a personal history of mental disorders were living in the northern part of Riyadh City. Thus, being born in urban places has not yet been established by studies to affect or predict the MHL of young people. Note that the participants in this work reported having a personal history of psychiatric mental disorders such as schizophrenia, shared psychiatric disorder, psychiatric disorder due to another medical condition, dissociative identity disorder, and postpartum psychiatric disorder. They also claimed to have neurotic mental disorders such as anxiety neurosis, eating disorder, postpartum depression, posttraumatic stress disorder, and obsessive-compulsive disorder. Additionally, they indicated having psychosomatic mental disorders such as acne, headache, allergy, arrhythmia, irritable colon, bronchial asthma, gastric ulcer, diabetes mellitus, and herpes ulcer. Among participants who reported personal mental health disorders (*n* = 40), majority (92.50%) claimed that they were either undiagnosed or unsure about having the disorder. The scarcity of reports of mental health disorders among participants could be because of not having any course related to mental health as the respondents were still registered in the second year (Level 4), thereby resulting in a lack of knowledge about mental health disorders. Being undiagnosed or unsure about having mental health disorders could also be influenced by Saudi culture where Islamic belief associated such disorders to spiritual and supernatural factors, predominantly reported among women (28). In addition, a study among Saudi patients reported that majority of the participants (*n* = 321) had no history of consulting mental health professional and sought treatment of their mental illness from faith healers (14). This finding could also be attributed to stigmatization and misleading information from the media (28). However, mental health services among adolescents in Saudi Arabia are available and delivered through mental health facilities including state universities (6). Hence, there is a need to provide educational interventions and enhance information campaign of the availability of mental health services in Saudi universities to increase awareness and early diagnosis of mental health disorders among university students.

Nonetheless, the findings contradicted a study conducted among university students in UK, for which many of the participants encountered mental health challenges and little was known about their general MHL and help-seeking behaviors. Additionally, the study revealed that participants with preceding mental disorders had increased reported scores of mental health knowledge than those with no history of mental disorders (29). Moreover, another study reported that achievements and absence of mental illness did not essentially translate into having mental wellness (27). In opposing results, nursing students from USA and China reported less frequent to frequent exposure to someone with mental illness, including family, friends and relatives (21). Furthermore, participants of another study who claimed to know someone with a mental disorder showed higher MHL scores than counterparts who knew no one with such a disorder (26).

Overall, the university students reported high levels of MHL. This finding was consistent with those of other studies that indicated high levels of MHL among participants (29, 46). However, O'Connor and Casey (46) revealed a contradictory finding indicating significant differences in MHL scores among mental health professionals and individuals with greater experience with mental health. In the current research, most participants were in their second year at the university and have not yet undertaken any course related to mental health. The participants reported that the MHL indicator with the first highest score was Item 17, thereby demonstrating that the participants were proficient in using a computer or telephone to obtain mental disorder data. This result was similar to that obtained by Gorczynski et al. (29) among UK university students. However, in a different study, the respondents gave the lowest score to items related to seeking help and information from general practitioners. These items were mentioned as being helpful but were not as highly rated as informal sources for first-aid support about MHL for depression (4). In particular, nursing students from USA and China shared comparable observations in managing depression and schizophrenia on a wide range of intervention choices comprising seeking professional help, psychotropic medications, and activity interventions (21). Furthermore, the study conducted by Byrne et al. (24) in Ireland showed that peers were depended on as a major foundation of emotional support among late adolescents and perceived specific accountabilities related to being a good friend. Nevertheless, most participants in the study investigated in Saudi Arabia by Mahfouz et al. (30) asserted that persons with mental illness were incapable of real friendships and that anyone can have a mental illness. By contrast, the lowest scored indicator in the current study was Item 22, which indicated that the university students disagreed with the notion that mental illness is not an actual medical challenge. In a seemingly opposing manner, Kutcher et al. (15) found that the majority of participants reported understanding that most persons with mental illness could live a normal life. This result may need further investigation to derive more conclusive results in the future.

The association between the participants' demographic characteristics of marital status, college attended, and academic level and their MHL revealed significant correlations. This finding may imply that university students who were single, studying in the Colleges of Nursing and Medicine, and registered in their second year (Level 4) at university had increased levels of MHL. This result was inconsistent with those of Salem et al. (36) in Riyadh, Saudi Arabia who found no meaningful relationship between the level of health literacy and the education level of their participants. However, Dias et al. (26) confirmed that socio-demographic variables such as gender and proximity to persons presenting with mental disorders were associated with a variation in MHL scores. In reference to gender differences, female participants showed higher scores than their male counterparts on the overall rating of their MHL. In another study, living in a metropolitan area was associated with help-seeking behavior from other professional sources (16).

This research has several limitations. The findings could not be generalized because the study focused on and included only female university students. The MHLS—Arabic version was also self-administered, thereby requiring students to show their own perception regarding their MHL. The data reported by students of having personal mental health disorders were reported by the participants as mostly undiagnosed or unsure, including the presence of such disorders in their families and relatives posing inability to verify the data with clinical records. As responses with four-point Likert were recoded to five-point Likert scale, it prohibited the participants to have an option for a neutral response. A mixed-method to include qualitative approach for this work could have enhanced and supported the quantitative reports of the students and thus may have contributed to limiting the generalizability of the findings. Additionally, a larger sample may have revealed a statistical association between the participants' demographic characteristics of age and place of residence and MHL. Finally, further research is needed to strengthen the usability of the MHLS—Arabic version.

The psychometric evaluation of the MHLS—Arabic version was robust and resulted in a valid and reliable scale. We utilized the Arabic version of the scale in our study and recommend its application with Arabic-speaking participants in the future. While at the university, students should enhance their MHL to overcome the challenges caused by the demanding tasks of university education. Mental health screening and assessment of MHL of students during enrolment to the university can promote awareness of their mental health status which can assist them utilize help-seeking behavior, family and social support, and coping strategies when needed.

This study has implications to academic and clinical practice. For university administrators and faculty, evaluating the information and perceptions toward mental health and illness of students as well as their MHL is necessary before accepting them to study. They must collaborate to plan and revise the policies and guidelines to support and enhance MHL not only among nursing students but also among other students in healthcare professions such as medicine and pharmacy. Culturally sensitive assessment of MHL can help university administrators develop programs that highlight the need for mental health knowledge interventions targeted at younger university students. Those schemes would improve the mental health of students while they continue their studies and in anticipation of the successful completion of their chosen programs.

For mental health professionals, the findings of this study can give information that there are university students with undiagnosed or unsure of having mental health disorders. Aside from enhancing educational measures and information campaign that mental health services are available in universities, necessary measures must be reinforced by mental health professionals so that students avail those services related to diagnosis, counseling, psychological therapy, and mental well-being. For future researchers, other factors that could affect MHL of university students must also be explored including family income, having mental health disorders, history of seeking medical help from mental health professional, Internet access, proximity to people presenting mental disorders, societal stigmatization, stress level, and sub-cultures based on geographical residence. When using the Arabic version of the MHL, future studies can also delve on its effect to the academic performance of both male and female students including those studying in non-health science colleges. We also recommend that future investigations will use the Arabic version of MHL, where no study has been conducted to date in national and international levels.

## Data Availability Statement

The raw data supporting the conclusions of this article will be made available by the authors, without undue reservation.

## Author Contributions

EA, DA, and RT: conceptualization. EA: data curation. ER: formal analysis. NA: investigation. EA and RT: methodology. EA, DA, and ER: project administration and validation. DA: supervision. EA, DA, NA, and RT: writing—original draft. EA, DA, ER, NA, and RT: writing—review and editing. All authors have read and agreed to the published version of the manuscript.

## Conflict of Interest

The authors declare that the research was conducted in the absence of any commercial or financial relationships that could be construed as a potential conflict of interest.

## Publisher's Note

All claims expressed in this article are solely those of the authors and do not necessarily represent those of their affiliated organizations, or those of the publisher, the editors and the reviewers. Any product that may be evaluated in this article, or claim that may be made by its manufacturer, is not guaranteed or endorsed by the publisher.
